# Outcomes of front-line tagraxofusp in patients with blastic plasmacytoid dendritic cell neoplasm (BPDCN) in a real-world scenario

**DOI:** 10.1186/s13045-026-01848-w

**Published:** 2026-10-07

**Authors:** Jan C. Schroeder, Philipp Faustmann, Snjezana Janjetovic, Anna Ossami Saidy, Hanna K. Zieger, Giuliano Filippini Velazquez, Thomas Melchardt, David Baden, Hristo Boyadzhiev, Tim Sauer, Christoph Schliemann, Jan-Henrik Mikesch, Niklas Gebauer, Susanne von Kroge, Paul J. Bröckelmann, Friederike Hildebrand, Veit Bücklein, Rebecca Wurm-Kuczera, Ulrich Keller, Thomas Pabst, Maher Hanoun, Claudia Lengerke, Evgenii Shumilov, Georg Lenz, Ulrike Bacher, Philipp Berning

**Affiliations:** 1 Department for Hematology, Oncology, Clinical Immunology and Rheumatology, University Hospital and Comprehensive Cancer Center Tübingen, Tübingen, Germany; 2 Department of Hematology and Cell Therapy, Helios Klinikum Berlin-Buch, Berlin, Germany; 3 Department of Hematology and Stem Cell Transplantation, West German Cancer Center and German Cancer Consortium (DKTK Partner Site Essen), University Hospital Essen, University of Duisburg-Essen, Essen, Germany; 4 Section for Stem Cell Transplantation, Augsburg University Hospital and Medical Faculty, Augsburg, Germany; 5 Department of Internal Medicine III with Haematology, Medical Oncology, Haemostaseology, Infectiology and Rheumatology, Cancer Research Laboratory of the Department of Internal Medicine III, Paracelsus Medical University, Salzburg, Austria; 6 Department of Internal Medicine II, University Hospital Schleswig-Holstein, Kiel, Germany; 7 University CancerCenter Schleswig-Holstein, University Hospital Schleswig-Holstein, Kiel, Germany; 8 Department of Medical Oncology, Inselspital, University Hospital Bern, University of Bern, Bern, Switzerland; 9 Habichtswald Hospital, Kassel, Germany; 10 Department of Internal Medicine V, University Hospital Heidelberg, Heidelberg, Germany; 11 Department of Medicine A, Hematology, Oncology and Pneumology, University Hospital Muenster, Muenster, Germany; 12 Department of Hematology and Oncology, University Hospital of Schleswig-Holstein, Lübeck, Germany; 13 Department of Oncology, Hematology and Bone Marrow Transplantation with Section Pneumology, University Cancer Center Hamburg, University Medical Center Hamburg-Eppendorf, Hamburg, Germany; 14 Department I of Internal Medicine, Center for Integrated Oncology Aachen Bonn Cologne Duesseldorf (CIO ABCD), Faculty of Medicine and University Hospital Cologne, University of Cologne, Cologne, Germany; 15 Department of Medicine III, LMU University Hospital, LMU Munich, Munich, Germany; 16 Department of Hematology, Oncology and Cancer Immunology, Charité - University Medical Center Berlin, Campus Benjamin Franklin, Berlin, Germany; 17 Department of Internal Medicine I, Klinikum Bremen Mitte, Bremen, Germany; 18 Department of Hematology and Central Hematology Laboratory, Inselspital, Bern University Hospital, University of Bern, Bern, Switzerland; 19 Department of Internal Medicine II, Hematology, Oncology, Clinical Immunology and Rheumatology, University Hospital Tübingen, Tübingen, Germany

**Keywords:** Blastic plasmacytoid dendritic cell neoplasm (BPDCN), Tagraxofusp, Bridging therapy, Allogeneic hematopoietic stem cell transplantation

## Abstract

Tagraxofusp (TAG), a CD123-directed protein-drug conjugate, is approved in front-line and relapsed/refractory blastic plasmacytoid dendritic cell neoplasm (BPDCN). Multicenter real-world outcomes and the contribution of up-front consolidation post-TAG remain less defined. We retrospectively analyzed 32 patients with BPDCN treated with frontline TAG (2019–2024) across 13 tertiary centers, contextualized against a historical cohort treated before TAG availability (*n* = 40). Patients were elderly (median age 71.5 years) with advanced disease, including skin (93.8%), bone marrow (84.4%), and peripheral blood involvement (53.1%). The overall response rate to TAG was 82.1%, with complete responses in 60.7%. Capillary leak syndrome occurred in 53.1% (grade ≥ 3 in 47.1%), with no unexpected safety signals. With a median follow-up of 18.4 months, 2-year overall survival (OS) and progression-free survival were 40.5% and 30.4%. Nearly half of patients proceeded to allogeneic stem cell transplantation post-TAG, associated with improved survival consistent with the primary analysis and landmark-adjusted analyses (HR 0.25 [0.06–1.14]; *p* = 0.073). Propensity score–matched comparison with the Non-TAG cohort showed comparable 2-year OS (59.4% vs 60.8%; *p* = 0.9). Frontline TAG was not associated with OS in pooled multivariable regression (HR 0.89 [0.37–2.15]; *p* = 0.803), while peripheral blood involvement remained associated with reduced OS (HR 2.63, 95% CI 1.1–6.27; *p* = 0.03). In this large multicenter real-world cohort, frontline TAG achieved high response rates with manageable toxicity. While unmeasured selection may contribute to the higher transplantation rate after TAG, durable remissions were achieved in patients undergoing post-TAG consolidation, supporting TAG as an induction and bridging strategy to allogeneic stem cell transplantation.

**Supplementary Information:**

The online version contains supplementary material available at https://doi.org/10.1186/s13045-026-01848-w.

**To the Editor,**

Blastic plasmacytoid dendritic cell neoplasm (BPDCN) is a rare, aggressive malignancy with a median age at diagnosis >65 years and poor outcomes with intensive chemotherapy [1–3]. The CD123-directed fusion protein tagraxofusp (TAG) has become first-line standard where available, with high response rates and a reversible toxicity profile dominated by capillary leak syndrome (CLS) [4, 5]. Durable disease control, however, requires consolidative allogeneic hematopoietic stem cell transplantation (allo-HSCT) [6, 7], and real-world data on TAG as a bridge to transplant remain scarce.

We retrospectively analyzed 32 patients treated with first-line TAG (11/2019–03/2024) across 13 German, Austrian, and Swiss tertiary centers and compared outcomes with a published historical cohort of 40 patients treated before TAG availability (2001–2022) [8]. Methodology is in the Supplementary Information.

Median age was 71.5 years (53.1% ≥70 years), 84.4% were male. Skin (93.8%), bone marrow (BM; 84.4%), and PB (53.1%) involvement were common; 15.6% had CNS involvement (Table 1; assessment at center discretion, Supplementary Information). Intrathecal prophylaxis was given to 40.0%, including 4 of 5 CNS-positive patients (data missing for one). CLS occurred in 53.1% (47.1% grade ≥ 3) and was more frequent in patients without BM involvement (Table S1). One-year non-relapse mortality was 15%, with no unexpected safety signals (Table S2, Figure S1). Best TAG response was CR in 60.7% and partial response in 21.4% of evaluable patients (Fig. 1A, Table S2); compartment-specific response was unavailable. Median follow-up was 18.4 months (IQR 8.8–32.9); median duration of response (DOR) was 7.8 months (range 0.2–61.4). Fourteen patients (43.8%) proceeded to frontline consolidative allo-HSCT (median time-to-transplant 80 days; one further patient underwent allo-HSCT later); reduced-intensity conditioning in 86.7% (Table S3).

**Table 1:** Baseline characteristics

Variable	Stratum	Value
Age at Diagnosis [years]	Median [Range]	71.5 [24.0, 87.0]
	<60	6 (18.8%)
	60–69	9 (28.1%)
	70–79	9 (28.1%)
	80-	8 (25%)
Gender	Female	5 (15.6%)
	Male	27 (84.4%)
ECOG Performance Score	0–1	27 (84.4%)
	≥2	3 (9.4%)
	Missing	2 (6.3%)
Skin manifestation	No	2 (6.3%)
	Yes	30 (93.8%)
Bone Marrow involvement	No	5 (15.6%)
	Yes	27 (84.4%)
Peripheral Blood	No	13 (40.6%)
	Yes	17 (53.1%)
	Missing	2 (6.3%)
Lymphadenopathy	No	14 (43.8%)
	Yes	18 (56.2%)
Hepatosplenomegaly	No	16 (50%)
	Yes	16 (50%)
CNS manifestation	No	26 (81.3%)
	Yes	5 (15.6%)
	Missing	1 (3.1%)
Allo-HSCT consolidation	No	17 (53.1%)
	Yes	15 (46.9%)
Time from diagnosis until Tagraxofusp [days]	Median [range]	20.5 [3.0, 262.0]
Total number of treatment lines	1	7 (21.9%)
	2	15 (45.9%)
	3	6 (18.7%)
	>3	4 (12.5%)
Median follow-up [months]	Median [IQR]	18.4 [8.8, 32.9]

**Fig. 1:**
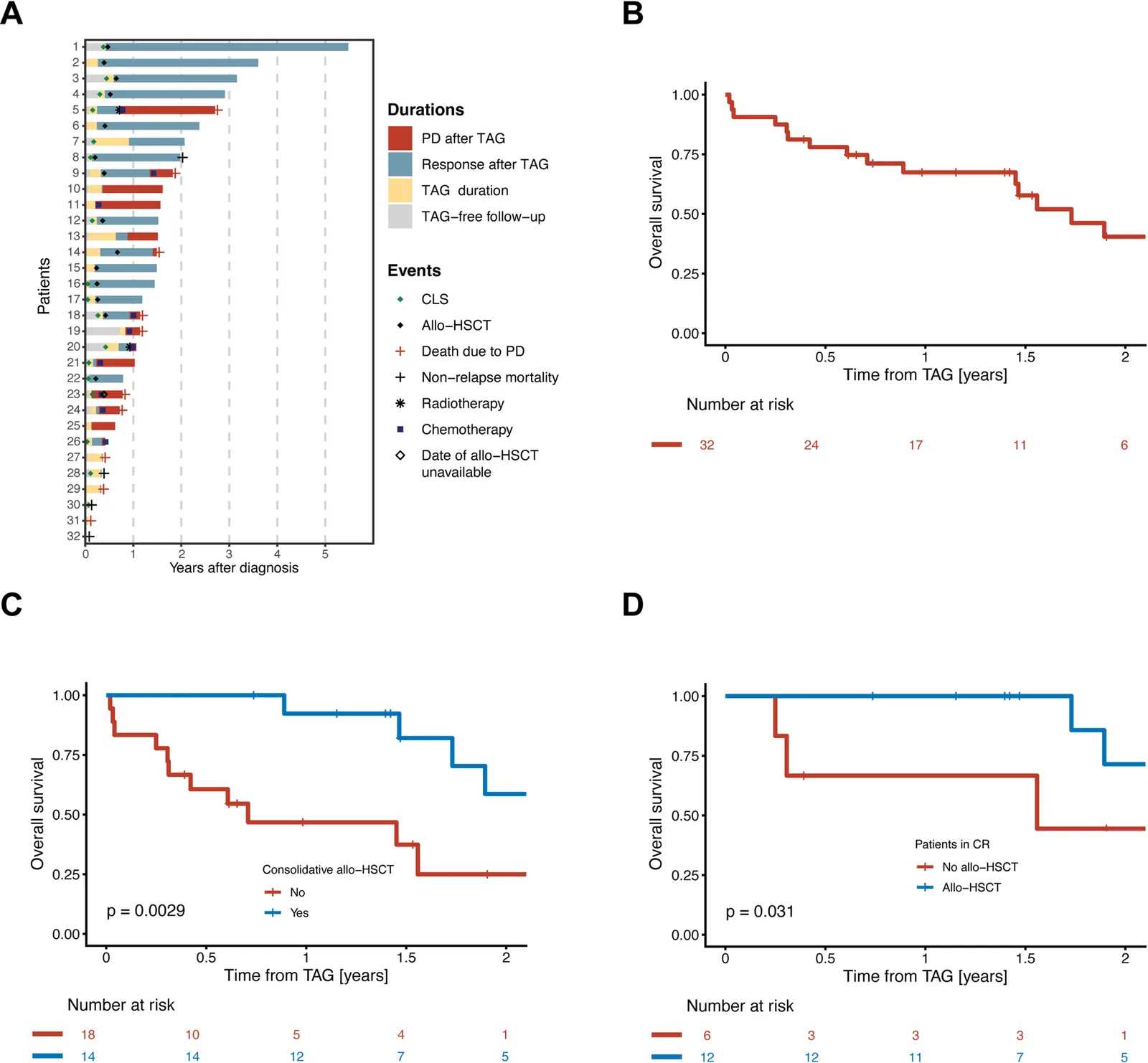
Tagraxofusp treatment outcomes. (**A**) Swimmer plot depicting tagraxofusp treatment duration, response duration, adverse events, subsequent treatment lines and consolidative treatments, and follow-up after first-line treatment with tagraxofusp. Kaplan-Meier estimated overall survival (OS) after tagraxofusp (TAG) treatment for the (**B**) whole cohort and (**C**) stratified by consolidative allogeneic hematopoietic stem cell transplantation (allo-HSCT). (**D**) OS among patients who achieved a complete response (CR), stratified by consolidative allo-HSCT

Two-year OS and PFS for the whole cohort were 40.5% and 30.4% (Fig. 1B, Figure S2). Transplanted versus non-transplanted patients had superior two-year OS (58.6% vs 24.9%, *p* = 0.003; Fig. 1C) and one-year PFS (92.9% vs 5.6%, *p* < 0.0001), with a comparable CR-restricted advantage (Fig. 1D). At last follow-up, 78.6% vs 22.2% remained in remission, and relapse occurred after a median of 504 days (range 245–632) vs 115 days (range 44–517). Two late CNS relapses occurred in transplanted patients; two-year OS was 48.1% in CNS-negative vs 0% in CNS-positive patients (*p* = 0.07, Figure S3).

Among 18 patients not proceeding to frontline allo-HSCT, reasons were mainly age/frailty or progression (Table S4); subsequent chemotherapy was associated with longer OS than venetoclax or no further therapy (Figure S4, Table S4).

The Non-TAG cohort was similar in age, sex, CNS and PB involvement but differed in skin, BM, and hepatosplenic involvement (Table S5). In pooled, adjusted analyses, treatment era was not associated with OS (HR 0.89 [0.37–2.15], *p* = 0.8), whereas PB involvement remained adverse (HR 2.63 [1.10–6.27], *p* = 0.03; Figure S5). Propensity-score matching (two-year OS 59.4% vs 60.8%, *p* = 0.9) and inverse probability weighting (HR 0.70 [0.32–1.51], *p* = 0.36) likewise showed no era-OS association (Table S6, Figure S6). Allo-HSCT was more frequent among TAG patients (43.8% vs 17.5%, *p* = 0.03). Time-dependent Cox regression (allo-HSCT, adjusted for age and PB), restricted to the TAG cohort where timing was available, suggested frontline allo-HSCT (HR 0.31 [0.06–1.52]; *p* = 0.15) and PB involvement (HR 2.64 [0.87–8.03]; *p* = 0.09, comparable to the pooled estimate) were not associated with OS in this small subgroup.

These findings suggest that any clinical benefit in the TAG era may not reflect a direct antileukemic advantage over conventional induction. Higher response rates and improved tolerability may have enabled more patients to reach consolidative allo-HSCT, an effect we cannot distinguish from unmeasured selection in patients who received TAG. A day-90 landmark analysis addressing time-to-transplant bias showed a trend toward improved OS with allo-HSCT (HR 0.25 [0.06–1.14], *p* = 0.073; *n* = 29, 13 events), though underpowered (Figure S7).

This multicenter real-world experience confirms favorable response rates and toxicity of frontline TAG [4, 5, 9, 10], supporting a bridge-to-transplant rather than curative role; higher CLS severity may reflect the elderly population and real-world management setting. Median DOR after TAG monotherapy was short (7.8 months), and durable remission remained largely limited to transplanted patients. The survival advantage of transplanted patients warrants caution, given non-randomized allocation; optimal CNS management requires dedicated study. Limitations include the retrospective design, a historical comparator subject to residual confounding (diagnostic approach, supportive care, transplant eligibility, unavailable ECOG/HCT-CI/molecular data), absent compartment-specific response assessment, and few events for multivariable modeling, although adjusted analyses yielded consistent conclusions. Overall, our data support TAG as an effective, well-tolerated induction strategy that may increase the proportion of BPDCN patients proceeding to allo-HSCT.

## Electronic supplementary material

Below is the link to the electronic supplementary material.

Supplementary Material 1

## Data Availability

No datasets were generated or analysed during the current study.
