# Supplementary material for: Outcomes of front-line tagraxofusp in patients with blastic plasmacytoid dendritic cell neoplasm (BPDCN) in a real-world scenario
Source: J Hematol Oncol. 2026 Oct 7;19:80. doi: 10.1186/s13045-026-01848-w (PMC13643986; doi:10.1186/s13045-026-01848-w)
Supplement: Supplementary file 1 [file 13045_2026_1848_MOESM1_ESM.docx]

**Supporting Information**

**Outcomes of front-line Tagraxofusp in patients with Blastic Plasmacytoid Dendritic Cell Neoplasm (BPDCN) in a real-world scenario**

**Supplementary Methods**

*Data collection*

This retrospective study included 32 consecutive patients treated with first-line TAG for BPDCN between November 2019 and March 2024 in 13 German/Austrian/Swiss tertiary centers. Clinical data were retrospectively extracted from medical records and electronic patient files. Outcomes of five patients reported in this analysis have previously been reported (18). In addition, a cohort of 40 patients with BPDCN previously reported by us and others (3) treated with conventional polychemotherapies before approval and availability of TAG at five European academic centers between 2001 and 2022 was used as a historical control (hereafter referred as Non-TAG cohort). The study was approved by the local ethics committee (Ethics Committee of the Westphalia-Lippe Medical Association no. 2023–071-f-S). All study procedures were performed in accordance with relevant guidelines, such as the Declaration of Helsinki and local regulations.

*Definitions and treatment*

Response to TAG was classified as complete response (CR), partial response (PR), stable disease (SD) and progressive disease (PD). Disease status was assessed by individual investigators according to standard criteria. Non-relapse mortality (NRM) was defined as death without prior relapse or disease progression following TAG treatment. Patients were monitored for CLS. CNS evaluation was not performed uniformly across all patients but was conducted based on clinical suspicion or evolving institutional standards. Intrathecal prophylaxis (methotrexate ± cytarabine and dexamethasone) was administered in selected patients, while patients with documented CNS involvement received therapeutic intrathecal treatment. Standard dosing of 12 ug/kg was used, except after dose reduction in indicated cases.

*Statistical analysis*

Continuous variables were summarized using medians and ranges or means and standard deviations, while categorical variables were described using frequencies and proportions. Differences between groups for categorical variables were assessed using Fisher’s exact test. Progression-free survival (PFS) and OS were calculated using Kaplan-Meier analyses from the date of TAG initiation. Differences in survival distributions were assessed using the log-rank test. Cumulative incidences of relapse and non-relapse mortality were estimated using competing-risk methods, with death in remission treated as a competing event for relapse. Associations between baseline patient or disease characteristics and OS were evaluated using univariate and multivariable Cox proportional hazards regression models, with results reported as hazard ratios (HRs) and 95% confidence intervals (CIs). To assess outcomes across treatment eras, pooled regression analyses including both the TAG-treated and Non-TAG cohorts were performed, with treatment era (TAG era vs Non-TAG era) included as an exposure variable and adjustment for relevant baseline prognostic factors. TAG-only regression analyses were conducted as exploratory analyses to identify prognostic factors within the TAG-treated population. To avoid immortal time bias, allo-HSCT was additionally modeled as a time-dependent covariate in a counting-process (start–stop) Cox formulation, in which each patient contributed a pre-transplant risk interval (allo-HSCT = 0) followed, for transplanted patients, by a post-transplant interval (allo-HSCT = 1). Variable selection for multivariable models was based on univariate significance (p<0.05) for age and PB involvement, while treatment era was retained as the exposure of interest. Multivariable analyses were restricted to complete cases for the included covariates. All tests were two-sided, and the type I error was fixed at 0.05 for factors associated with time-to-event outcomes. All analyses were performed using R statistical software version 4.5.2 (available online at http://www.R-project.org).

**Supplementary Figure Legends**

**Figure S1. Competing risk analysis of non-relapse mortality and relapse after tagraxofusp**

Cumulative incidences of non-relapse mortality (NRM) and relapse were estimated using competing-risk methods, with death in remission treated as a competing event for relapse. TAG=tagraxofusp.

**Figure S2. Tagraxofusp progression-free survival**

Kaplan-Meier estimated progression-free survival (PFS) after Tagraxofusp (TAG) treatment for the **(A)** whole cohort and **(B)** stratified by consolidative allogeneic hematopoietic stem cell transplantation (allo-HSCT). PFS among patients who achieved a **(C)** complete response (CR), stratified by consolidative allo-HSCT.

**Figure S3. Overall survival by baseline CNS involvement**

Kaplan-Meier-estimated overall survival (OS) from TAG initiation, stratified by baseline CNS involvement, n=32. Two late CNS relapses occurred (days 504, 632 from TAG initiation), both in transplanted patients; one occurred despite intrathecal prophylaxis in a patient with baseline CNS involvement.

**Figure S4. Overall survival by subsequent therapy in patients without frontline allo-HSCT**

Kaplan-Meier-estimated overall survival (OS) from TAG initiation, stratified by subsequent-therapy category (any intensive chemotherapy, venetoclax ± hypomethylating agent, salvage allo-HSCT, or none). n=17 evaluable, excluding one patient with ongoing frontline TAG without relapse.

**Figure S5. Pooled regression analysis to compare TAG outcomes with historical cohort**

Regression was performed on the pooled dataset containing 32 tagraxofusp first-line (TAG) patients and 40 patients from a historical, non-TAG treated cohort. TAG era, i.e. TAG cohort vs. historical cohort, was used as a covariate together with the shown baseline variables and included in uni- and multivariate regression. Multivariate analyses were restricted to complete cases for the included covariates and had 24 events per 3 variables.

**Figure S6. Propensity-score-matched comparison of overall survival between TAG and Non-TAG cohorts, stratified by consolidative allo-HSCT**

Kaplan-Meier estimates of overall survival (OS) for the propensity-score-matched TAG and Non-TAG cohorts (n=17 per cohort), matched on age at diagnosis, bone marrow involvement, and skin involvement. Top: OS in the overall matched cohort (p=0.93, log-rank test). Middle: OS in matched patients who received consolidative allo-HSCT after frontline therapy (n=9 TAG vs. n=5 Non-TAG; p=0.29). Bottom: OS in matched patients who did not receive consolidative allo-HSCT (n=8 TAG vs. n=12 Non-TAG; p=0.51). Numbers at risk are shown below each panel; tick marks indicate censored patients.

**Figure S7. Landmark analysis of overall survival by allo-HSCT status**

Landmark analysis for overall survival (OS) among 29 patients who were alive at 90 days after initiation of tagraxofusp. Allo-HSCT status was defined based on whether transplantation had occurred prior to the landmark. During follow-up, 13 events occurred in this cohort.

**Figure S1. Competing risk analysis of non-relapse mortality and relapse after tagraxofusp**

**Figure S2. Tagraxofusp progression-free survival**

**Figure S3. Overall survival by baseline CNS involvement**

**Figure S4. Overall survival by subsequent therapy in patients without frontline allo-HSCT**

**Figure S5. Pooled regression analysis to compare TAG outcomes with historical cohort**

**Figure S6. Propensity-score-matched comparison of overall survival between TAG and Non-TAG cohorts, stratified by consolidative allo-HSCT**

**Figure S7. Landmark analysis of overall survival by allo-HSCT status**

**Supplementary Tables**

**Table S1: Capillary leak syndrome characteristics**

| **Variable** | **Stratum** | **Value** |
| --- | --- | --- |
| CLS grade (n=17) | Grade 1 | 1 (5.9%) |
|  | Grade 2 | 8 (47.1%) |
|  | Grade 3 | 4 (23.5%) |
|  | Grade 4 | 4 (23.5%) |
| CLS onset (n=17) | Cycle 1 | 15 (88.2%) |
|  | Cycle 2 | 8 (11.8%) |
| Cycle-1 interruption | Among CLS (n=17) | 5 (29.4%) |
|  | Among non-CLS, evaluable (n=14) | 3 (21.4%) |
| Dose-reduction | Yes | 2 (6.2%) |
|  | No | 30 (93.8%) |
| CLS by BM involvement | BM-negative (n=5) | 5 (100%) |
|  | BM-positive (n=27) | 13 (44.4%); p=0.046 |

**Table S2: Tagraxofusp treatment response and toxicities**

| **Variable** | **Stratum** | **Value** |
| --- | --- | --- |
| Best Response to Tagraxofusp | CR | 17 (53.1%) |
|  | PR | 6 (18.8%) |
|  | SD | 2 (6.3%) |
|  | PD | 3 (9.4%) |
|  | Missing | 4 (12.5%) |
| Median Duration of Response [months] | Overall | 7.8 [0.2, 61.4] |
| First Cycle of Tagraxofusp administered completely | No | 8 (25.0%) |
|  | Yes | 23 (71.9%) |
|  | Missing | 1 (3.1%) |
| Days required for application of first cycle | Overall | 5.0 [2.0, 14.0] |
| Tumor Lysis Syndrome | Grade 0 | 7 (21.9%) |
|  | Grade 3 | 5 (15.6%) |
|  | Grade 4 | 4 (12.5%) |
|  | Missing | 16 (50.0%) |
| Capillary leak syndrome | No | 15 (46.9%) |
|  | Yes | 17 (53.1%) |
| Albumin Substitution during Tagraxofusp treatment | No | 8 (25.0%) |
|  | Yes | 22 (68.8%) |
|  | Missing | 2 (6.3%) |
| Steroid usage during Tagraxofusp treatment | No | 7 (21.9%) |
|  | Yes | 22 (68.8%) |
|  | Missing | 3 (9.4%) |
| ALT increased CTCAE ≥3 | No | 18 (56.3%) |
|  | Yes | 7 (21.9%) |
|  | Missing | 7 (21.9%) |
| AST increased CTCAE ≥3 | No | 10 (31.3%) |
|  | Yes | 15 (46.9%) |
|  | Missing | 7 (21.9%) |
| Bilirubin increased CTCAE ≥3 | No | 24 (75.0%) |
|  | Yes | 2 (6.3%) |
|  | Missing | 6 (18.8%) |
| Thrombocytopenia CTCAE ≥3 | No | 10 (31.3%) |
|  | Yes | 19 (59.4%) |
|  | Missing | 3 (9.4%) |

**Table S3: Allo-HSCT conditioning, donor type and graft source**

| **Variable** | **Stratum** | **Value** |
| --- | --- | --- |
| Allo-HSCT conditioning intensity | MAC | 2 (13.3%) |
|  | RIC | 13(86.7%) |
| TBI as conditioning | No | 3 (23.1%) |
|  | Yes | 10 (76.9%) |
|  | Missing | 3 |
| Donor Type | MUD | 12 (80%) |
|  | MMUD | 0 (0%) |
|  | MRD | 1 (6.7%) |
|  | Haplo | 2 (13.3%) |
| HCT-CI | 0 | 2 (16.7%) |
|  | 1-2 | 4 (33.3%) |
|  | ≥3 | 6 (50%) |
|  | Missing | 3 |
| Graft source | PB | 13 (100%) |
|  | BM | 0 (0%) |
|  | Missing | 2 |

**Table S4: Patients not undergoing allo-HSCT: reasons and subsequent therapy.**

Category totals (n=18): age/frailty, 6 (33.3%); refractory disease/progression, 8 (44.4%); unrelated fatal event, 2 (11.1%); fatal toxicity and patient preference, 1 each (5.6%).

Subsequent therapy: chemotherapy, 6 (33.3%); venetoclax ± hypomethylating agent, 6 (33.3%; 5/6 with a hypomethylating agent); salvage allo-HSCT, 1 (5.6%); none, 4 (22.2%); ongoing frontline TAG, 1 (5.6%). Median OS and 2-year OS by subsequent-therapy category are shown in Figure S4.

| **Patient** | **Reason for no frontline allo-HSCT** | **Subsequent therapy** | **OS (days)** | **Vital status** |
| --- | --- | --- | --- | --- |
| Pt 1 | Progression | Venetoclax + AZA | 143 | Alive |
| Pt 4 | Progression | Venetoclax + AZA | 222 | Deceased |
| Pt 5 | Patient preference | None | 112 | Deceased |
| Pt 8 | Age/frailty (87y) | Chemotherapy | 359 | Alive |
| Pt 10 | Age/frailty (86y) | Chemotherapy | 239 | Alive |
| Pt 13 | Unrelated fatal event | None | 7 | Deceased |
| Pt 14 | Progression | Venetoclax + AZA | 569 | Deceased |
| Pt 15 | Unrelated fatal event | None | 91 | Deceased |
| Pt 16 | Progression | Chemotherapy | 962 | Deceased |
| Pt 20 | Fatal toxicity | None | 12 | Deceased |
| Pt 21 | Progression | Chemotherapy | 15 | Deceased |
| Pt 23 | Progression | Chemotherapy | 560 | Alive |
| Pt 24 | Progression (refractory) | Salvage allo-HSCT (in PD) | 259 | Deceased |
| Pt 25 | Age/frailty (82y) | Venetoclax + AZA | 114 | Deceased |
| Pt 27 | Age/frailty (84y) | Ongoing TAG, CR | 696 | Alive |
| Pt 29 | Progression | Venetoclax | 154 | Deceased |
| Pt 30 | Progression | Venetoclax + decitabine | 530 | Deceased |
| Pt 33 | Age/frailty (84y) | Chemotherapy | 224 | Alive |

**Table S5: Comparison of baseline variables in the Tagraxofusp and historical BPDCN cohorts**

| **Parameter** | **TAG (n=32)** | **Historical cohort (n=40)** | **p-value** |  |
| --- | --- | --- | --- | --- |
|  |  |  |  |  |
| **Age at Diagnosis [years]** |  |  |  |  |
| Median [Min, Max] | 71.5 [24.0, 87.0] | 69.5 [15.0, 91.0] | 0.676 |  |
| **Gender** |  |  |  |  |
| Female | 5 (15.6%) | 10 (25.0%) | 0.496 |  |
| Male | 27 (84.4%) | 30 (75.0%) |  |  |
| **Skin manifestation** |  |  |  |  |
| No | 2 (6.3%) | 15 (37.5%) | 0.00475 |  |
| Yes | 30 (93.8%) | 25 (62.5%) |  |  |
| **Bone Marrow involvement** |  |  |  |  |
| No | 5 (15.6%) | 21 (52.5%) | 0.00279 |  |
| Yes | 27 (84.4%) | 19 (47.5%) |  |  |
| **Peripheral Blood** |  |  |  |  |
| No | 13 (43.3%) | 10 (58.8%) | 0.473 |  |
| Yes | 17 (56.7%) | 7 (41.2%) |  |  |
| Missing | 2 | 23 |  |  |
| **Lymphadenopathy** |  |  |  |  |
| No | 14 (43.8%) | 25 (62.5%) | 0.177 |  |
| Yes | 18 (56.3%) | 15 (37.5%) |  |  |
| **Hepatosplenomegaly** |  |  |  |  |
| No | 16 (50.0%) | 35 (87.5%) | 0.00129 |  |
| Yes | 16 (50.0%) | 5 (12.5%) |  |  |
| **CNS manifestation** |  |  |  |  |
| No | 27 (84.4%) | 37 (92.5%) | 0.476 |  |
| Yes | 5 (15.6%) | 3 (7.5%) |  |  |
| **Consolidative allo-HSCT** |  |  |  |  |
| No | 18 (56.3%) | 33 (82.5%) | 0.03 |  |
| Yes | 14 (43.7%) | 7 (17.5%) |  |  |

**Table S6:** **Comparison of baseline variables in the Tagraxofusp and historical BPDCN cohorts after propensity-score matching for age at diagnosis, bone marrow involvement, and skin involvement**

| **Parameter** | **TAG (n=17)** | **Historical cohort (n=17)** | **p-value** |  |
| --- | --- | --- | --- | --- |
|  |  |  |  |  |
| **Age at Diagnosis [years]** |  |  |  |  |
| Median [Min, Max] | 68.0 [46.0, 86.0] | 68.0 [35.0, 83.0] | 0.657 |  |
| **Gender** |  |  |  |  |
| Female | 3 (17.6%) | 2 (11.8%) | 1 |  |
| Male | 14 (82.4%) | 15 (88.2%) |  |  |
| **Skin manifestation** |  |  |  |  |
| No | 2 (11.8%) | 3 (17.6%) | 1 |  |
| Yes | 15 (88.2%) | 14 (82.4%) |  |  |
| **Bone Marrow involvement** |  |  |  |  |
| No | 5 (29.4%) | 4 (23.5%) | 1 |  |
| Yes | 12 (70.6%) | 13 (76.5%) |  |  |
| **Peripheral Blood** |  |  |  |  |
| No | 9 (52.9%) | 5 (50.0%) | 1 |  |
| Yes | 8 (47.1%) | 5 (50.0%) |  |  |
| Missing | 0 | 7 |  |  |
| **Lymphadenopathy** |  |  |  |  |
| No | 7 (41.2%) | 10 (58.8%) | 0.493 |  |
| Yes | 10 (58.8%) | 7 (41.2%) |  |  |
| **Hepatosplenomegaly** |  |  |  |  |
| No | 10 (58.8%) | 14 (82.4%) | 0.259 |  |
| Yes | 7 (41.2%) | 3 (17.6%) |  |  |
| **CNS manifestation** |  |  |  |  |
| No | 14 (82.4%) | 14 (82.4%) | 1 |  |
| Yes | 3 (17.6%) | 3 (17.6%) |  |  |
| **auto-HSCT** |  |  |  |  |
| No | 17 (100%) | 13 (76.5%) | 0.11 |  |
| Yes | 0 (0%) | 4 (23.5%) |  |  |
| **Consolidative allo-HSCT** |  |  |  |  |
| No | 8 (47.1%) | 14 (82.4%) | 0.143 |  |
| Yes | 9 (52.9%) | 3 (17.6%) |  |  |
